# Tooth Loss as a Predictor of Long-Term Care Requirements in the Elderly: A Study in Kobe City, Japan

**DOI:** 10.7759/cureus.49851

**Published:** 2023-12-02

**Authors:** Yasumasa Kakei, Tatsuo Kagimura, Yasuji Yamamoto, Tohmi Osaki, Hiroyuki Kajita, Shinsuke Kojima, Hisatomo Kowa, Miyuki Kawabata, Takumi Hasegawa, Masaya Akashi, Yoji Nagai

**Affiliations:** 1 Department of Oral and Maxillofacial Surgery, Kobe University Hospital, Kobe, JPN; 2 Translational Research Centre for Medical Innovation, Foundation for Biomedical Research and Innovation, Kobe, JPN; 3 Department of Biosignal Pathophysiology, Kobe University Graduate School of Medicine, Kobe, JPN; 4 Faculty of Rehabilitation, Kobe Gakuin University, Kobe, JPN; 5 Department of Rehabilitation Science, Kobe University Graduate School of Health Sciences, Kobe, JPN; 6 Department of Clinical Research Facilitation, Kyoto University Hospital, Kyoto, JPN

**Keywords:** body mass index, frailty, long-term care need, quality of life, tooth loss

## Abstract

Introduction: The Kobe project, which utilizes prospective data from the national health insurance system, focuses on early detection and preventive strategies through the Frail Kenshin health check-up program. Previous research has underscored the correlation between tooth loss and the decline in physical and cognitive functions. In this study, using Kobe project data, we examined the link between remaining teeth and long-term care needs in individuals aged 64-65 years, with primary and secondary objectives involving various health parameters and quality of life.

Methods: We analyzed baseline data from a prospective study conducted alongside the Frail Check program for generally healthy individuals aged 64-65 years to examine the relationship between the number of remaining teeth and various health indicators. This study focused on citizens aged 64-65 years to identify those at risk of needing long-term care by the age of 65 years.

Results: Data from 1,530 participants were obtained, excluding eight individuals for specific reasons. At the end of the follow-up period, 41 (2.7%) individuals required support and 15 (1.0%) needed long-term care alone. The data revealed a significant association between the number of remaining teeth and the need for long-term care or support, as demonstrated by the Cochran-Armitage trend test (p<0.001).

Although trends were noted for nutrition and total Cognitive Functional Instrument Self scores, they did not reach statistical significance. Additionally, a decrease in the number of remaining teeth was significantly associated with worse European Quality of Life Five Dimensions (EQ-5D-5L) visual analog scale scores, mobility, and regular activities (p<0.001).

Conclusion: Tooth loss indicates the potential long-term care needs of older adults. Monitoring oral health is crucial for addressing care requirements.

## Introduction

Physical and cognitive decline necessitates the provision of long-term care and support services for community-dwelling older adults. As Japan currently has the largest population of super-aged individuals worldwide [1], care for this population places a serious burden on the healthcare system and has wide-ranging socioeconomic implications [2,3].

Various population-based studies have evaluated methods for the prevention and early detection of physical and cognitive decline in community-dwelling older adults in Japan. One such initiative is the Kobe project, which includes the first large prospective study to evaluate the predictive ability of neuropsychological assessment tools for long-term care certification in older adults [4]. One of the studies in this project used the data collected by local government municipal offices from older adults enrolled in the Kokumin Kenko Hoken National Health Insurance system in Japan; the insurance covers all Japanese citizens and long-term residents. In this context, Frail Kenshin is a health check-up program conducted by Kobe City that includes physically accessible components in addition to self-assessed questions related to frailty. It evaluates motor and oral functions, nutrition, forgetfulness, mental and dental health, chewing ability, swallowing ability, grip strength, height, weight, body mass index (BMI), and other health-related parameters.

Numerous epidemiological studies have reported an association between tooth loss and physical and cognitive decline in older adults [5-8]. Reports suggest that in addition to a reduction in the number of food items that can be consumed owing to a decrease in the number of chewing points, a decrease in bite strength leads to a decline in motor skills, including grip strength and walking function, which are also associated with a decline in cognitive function [9-11]. A study using nationwide data from Korea also showed an association between the number of remaining teeth and health-related quality of life (QOL) [12].

In view of these findings, we analyzed data obtained from a prospective study under the Kobe project. The study evaluated the relationship between data obtained using the Cognitive Functional Instrument (CFI), European Quality of Life Five Dimensions (EQ-5D-5L), and other frailty-related parameters and explored the risk of future long-term care and support needs within three years in older adults aged 64-65 years in the assessment year.

The primary objective of this study was to assess the association between the number of teeth and the need for long-term care. The secondary objectives were to assess the association between the number of teeth and chewing points, grip strength, motor functions such as walking, and QOL.

This article was previously posted to the Research Square preprint server on 27 July 2022.

## Materials and methods

Ethical issues

All participants were treated in accordance with the principles of the Declaration of Helsinki developed by the World Medical Association in 1964 and modified in October 2013 at the World Medical Association Fortaleza General Assembly (Brazil) [13]. All research conformed to the “Law on Protection of Personal Information” (May 30, 2003; Law No. 57) [14]. This study was approved by the Ethics Review Committee of the WHO (ERC.0002899) and Kobe University (Approval No. 170018). The need for informed consent was waived as this was a post hoc analysis of the data obtained from a prospective study.

Study design and setting

This study analyzed baseline data from a prospective study conducted in conjunction with the Frail Check program for Kobe citizens aged 64-65 years, initiated by the municipal office of Kobe City. The Frail Check program, which aims to assess general health and frailty-related parameters, incorporates dental assessments in its evaluation process.

Participants aged 64-65 years were selected because they represented the minimum age of the elderly in Japan. Focusing on this age group, we aimed to identify individuals at risk of requiring long-term care at 65 years of age. Early intervention is crucial for preventing or minimizing the need for long-term care, particularly in individuals with fewer remaining teeth who are at higher risk.

Baseline data were obtained between August 2017 and March 2018. The program was conducted repeatedly at multiple venues within the community. Numerous subjects attended each occasion where various general health and frailty-related parameters, including dental assessments, were individually evaluated. Following the assessments, the subjects received their results along with medical and non-medical recommendations.

Long-term care insurance data up to March 31, 2020, were obtained from the administrative records of Kobe City, allowing for a comprehensive analysis of the association between the number of teeth and the need for long-term care.

By incorporating dental assessments within the Frail Check program, specific relationships between the number of teeth and the need for long-term care were identified, and secondary objectives were explored, such as the association between the number of teeth and variables including the number of chewing points, grip strength, motor functions (e.g., walking), and QOL.

Subjects

The subjects included citizens of Kobe, Japan, aged between 64 and 65 years who attended the Frail Check program in Kobe City. Hyogo Prefecture, where Kobe City is located, is representative of the national average with respect to the number of elderly people needing long-term care. Citizens who were already certified for long-term care at the time of the Frail Check data collection were excluded.

Variables evaluated

Data regarding the Frail Check test, CFI Self questionnaire, and EQ-5D-5L assessments were collected during the Frail Check program conducted in Kobe. The Frail Check sheet devised by Kobe City examines the following data: age, sex, daily living activity/forgetfulness, exercise function, nutritional status, oral and dental and cardiac health, calf circumference, results of the finger-ring (Yubi-wakka) test (using fingers of both hands to make a ring around one’s calf), chewing and grip strength, swallowing, and stand-up tests.

As described by Nagai et al. [4], details pertaining to the number of remaining teeth were obtained from the Frail Check data for this analysis; the mastication score and number of foods that could be chewed were also assessed; the mastication score and the number of food items that could be chewed were also assessed. The remaining teeth were categorized into five groups: 0, 1-4, 5-9, 10-19, and >20. These categories were retained for this analysis.

Tools used in the prospective study

CFI Self Questionnaire Japanese Version

The Japanese version of the CFI Self questionnaire was used to evaluate cognitive function-related ADL (activities of daily living) in the prospective study. The questionnaire consists of 14 items, where “yes” = 1 point, “probable” = 0.5 points, and “no” = 0 points; the total of the points for all items constitutes the individual scores.

EQ-5D-5L for Evaluating Health-Related QOL

EQ-5D-5L was used for measuring health-related QOL; with this tool, individual QOL is defined as a single value between 0 and 1 with upper and decreased limits of “complete health = 1” and “death = 0,” respectively, based on answers to five questions (five levels).

Information regarding long-term care certification

Information on subjects certified as needing long-term care within three years was obtained from the Long-Term Care Certification database of Kobe City. In accordance with the Long-Term Care Insurance Law standard, the Kobe City Long-Term Care Need Certification criteria evaluated the following: level of long-term care needed, scored between 1 and 5 based on the assessment of care requirements, and level of assistance needed, scored as 1 or 2.

Sample size

The Frail Check data for 1530 subjects from Kobe City were obtained for this study. Among the eight individuals excluded, five had moved out of Kobe City; therefore, the results of the Kobe City-approved certification for the need for long-term care or support were unknown. Two cases were excluded because of duplication of Frail Check results, and one patient was already certified as requiring long-term care at the time of Frail Check screening.

Statistical analysis

We performed a trend test for the number of teeth for each evaluation indicator. Dichotomous variables, such as the presence or absence of care needs, were evaluated using the Cochran-Armitage trend test, which determines the proportion of individuals requiring care depending on the number of remaining teeth. Continuous variables and ordinal data were evaluated using the Jonckheere-Terpstra test. For this analysis, p-values <0.001 were considered statistically significant. SAS version 9.4 (SAS Institute Inc., Cary, NC) and R (Version 3.6; R Core Team 2020) were used for the analyses.

## Results

The data of 1508 older adults were analyzed in this study; there were 957 (63.5%) and 546 (36.2%) female and male participants, respectively. At the end of the follow-up period, 41 (2.7%) and 15 (1.0%) individuals were certified as needing support or long-term care and long-term care alone, respectively.

The Cochran-Armitage trend test demonstrated a significant increase in the need for long-term care or support, with a progressive decline in the number of remaining teeth (p<0.001). Certification for long-term care needs also followed a similar trend (Table 1).

**Table 1:** Association between long-term care need or long-term care and support need and the number of remaining teeth, as indicated by the Frail Check data

			Number of Teeth (n)										Cochran-Armitage trend test
	Total		0		1-4		5-9		10-19		>20		
	n	%	n	%	n	%	n	%	n	%	n	%	
Total	1,508	100	17	100	18	100	39	100	176	100	1,258	100	
Gender													
Female	957	63.5	6	35.3	7	38.9	21	53.8	91	51.7	832	66.1	
Male	546	36.2	11	64.7	11	61.1	18	46.2	85	48.3	421	33.5	
Information not provided	5	0.3	0	0	0	0	0	0	0	0	5	0.4	
Long-term care or support need													
Yes	41	2.7	4	23.5	0	0	5	12.8	9	5.1	23	1.8	p<0.001
No	1,467	97.3	13	76.5	18	100	34	87.2	167	94.9	1,235	98.2	
Long-term care need													
Yes	15	1	1	5.9	0	0	2	5.1	5	2.8	7	0.6	p=0.004
No	1,493	99	16	94.1	18	100	37	94.9	171	97.2	1,251	99.4	

**Figure 1:**
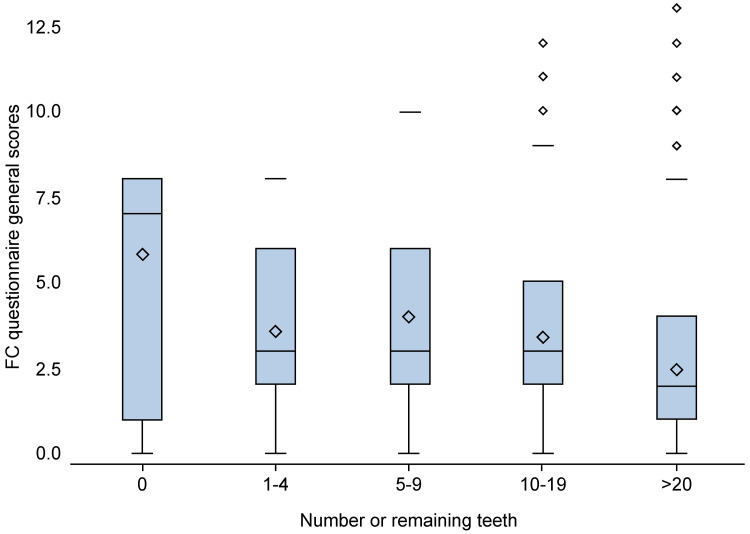
Box plot showing the declining trend in general function, as assessed by the Frail Check questionnaire

Evaluation of the Frail Check data demonstrated that a decrease in the number of remaining teeth was associated with a decline in general frailty, including motor and oral functions, prevention of seclusion, memory, mental health, mastication scores, total number of food items that could be chewed, and BMI (p<0.001) (Figure 1). In addition, a reduction in the number of remaining teeth was associated with decreased calf and standing test scores during the check-up (p<0.001). Although similar trends were noted for the nutrition and total CFI Self scores, these associations were not statistically significant (Tables 2, 3). Analysis of the EQ-5D-5L utility index data demonstrated that a reduction in the number of remaining teeth was associated with a significant worsening of EQ-5D-5L visual analog scale scores, degree of mobility, and regular activities (p<0.001) (Table 4).

**Table 2:** Data pertaining to items in the Frail Check questionnaire that demonstrated association with the number of remaining teeth FC, Frail Check; CFI, cognitive functional instrument; BMI, body mass index; SD, standard deviation.

			Number of teeth (n)					Jonckheere- Terpstra test
		Total	0	4-Jan	9-May	19-Oct	>20	
FC questionnaire Comprehensive score	N	1,508	17	18	39	176	1,258	
	Mean	2.66	5.82	3.56	4	3.4	2.45	p<0.001
	SD	2.28	4.59	2.33	2.62	2.4	2.13	
FC questionnaire Motor function score	N	1,508	17	18	39	176	1,258	
	Mean	0.74	1.71	0.83	1.15	1	0.68	p<0.001
	SD	1.01	1.72	1.04	1.01	1.12	0.96	
FC questionnaire Nutrition	N	1,508	17	18	39	176	1,258	
	Mean	0.35	0.35	0.33	0.38	0.32	0.35	p=0.863
	SD	0.52	0.49	0.49	0.49	0.48	0.53	
FC questionnaire Oral function score	N	1,508	17	18	39	176	1,258	p<0.001
	Mean	0.62	0.76	1.06	1.03	0.9	0.56	
		Number of teeth (n)						Jonckheere- Terpstra test
		Total	0	4-Jan	9-May	19-Oct	>20	
FC questionnaire Prevention of withdrawal score	N	1,508	17	18	39	176	1,258	
	Mean	0.15	0.41	0.33	0.26	0.21	0.13	p<0.001
	SD	0.38	0.62	0.49	0.44	0.42	0.36	
FC questionnaire Forgetfulness score	N	1,508	17	18	39	176	1,258	
	Mean	0.36	1.18	0.5	0.54	0.45	0.33	p<0.001
	SD	0.61	1.13	0.71	0.68	0.66	0.58	
FC questionnaire Mental health score	N	1,508	17	18	39	176	1,258	
	Mean	0.64	2.24	1.22	1.05	1.03	0.55	p<0.001
	SD	1.2	2.05	1.63	1.54	1.48	1.09	
FC questionnaire Chewing test score	N	1,508	17	18	39	176	1,258	
	Mean	48.22	38.18	41.5	43.54	46.34	48.86	p<0.001
	SD	4.44	14.85	8.28	6.27	5.04	3.41	
Total number of foods that can be chewed (1 point: chewable 0.5 points: hard to chew)	N	1,506	17	18	39	176	1,256	
	Mean	24.14	19.09	20.75	21.77	23.17	24.47	p<0.001
		Number of teeth (n)						Jonckheere- Terpstra test
		Total	0	4-Jan	9-May	19-Oct	>20	
	SD	2.04	7.43	4.14	3.13	2.52	1.4	
Total CFI Self score	N	1,451	16	16	39	171	1,209	p=0.007
	Mean	1.86	3.5	2.47	2.03	2.25	1.77	
	SD	1.88	3.49	2.57	1.94	2.22	1.77	
FC questionnaire Calf circumference	N	1,507	17	18	39	176	1,257	p<0.001
	Mean	35	36.1	35.3	35.9	35.8	34.9	
	SD	3.2	3.1	3.3	3.3	3.4	3.2	
FC questionnaire Standing up examination score	N	1,467	15	17	38	167	1,230	
	Mean	9.27	8.2	8.24	8.68	8.79	9.38	p<0.001
	SD	2.59	2.46	2.44	2.69	2.26	2.62	
FC questionnaire BMI	N	1,445	15	17	39	172	1,202	
	Mean	22.58	24.46	23.19	24.14	23.7	22.34	p<0.001
	SD	3.4	4.5	2.54	4.09	3.69	3.28	
FC questionnaire Mastication	N	1,408	11	11	29	150	1,207	
		Number of teeth (n)						Jonckheere- Terpstra test
		Total	0	4-Jan	9-May	19-Oct	>20	
	Mean	2.35	2.91	2.82	2.62	2.5	2.31	p=0.004
	SD	0.99	1.14	1.08	1.29	1.06	0.97	
FC questionnaire Right grip	N	1,485	17	17	39	172	1,240	
	Mean	27.8	27.9	32.5	28.5	28.9	27.5	p=0.019
	SD	8.6	9.1	9.7	8.9	9.3	8.4	
FC questionnaire Left grip	N	1,470	17	17	39	171	1,226	
	Mean	26.7	27.6	31.6	26.5	28.1	26.5	p=0.020
	SD	8.4	8.8	9.7	8.8	9.6	8.2	

**Table 3:** Data pertaining to assessed items in the Frail Check questionnaire that demonstrated an association with the number of remaining teeth FC, Frail Check.

	Total		Number of teeth (n)										Jonckheere -Terpstra test
			0		1～4		5～9		10～19		>20		
	n	%	n	%	n	%	n	%	n	%	n	%	
Total	1,508	100	17	100	18	100	39	100	176	100	1,258	100	
FC questionnaire/Grip strength assessment													p=0.029
Weak	22	1.5	2	11.8	0	0.0	2	5.1	4	2.3	14	1.1	
Somewhat weak	176	11.7	3	17.6	1	5.6	6	15.4	24	13.6	142	11.3	
Normal	230	15.3	2	11.8	4	22.2	7	17.9	27	15.3	190	15.1	
Somewhat strong	789	52.3	9	52.9	9	50.0	18	46.2	90	51.1	663	52.7	
Strong	274	18.2	1	5.9	3	16.7	6	15.4	28	15.9	236	18.8	
Not performed	17	1.1	0	0.0	1	5.6	0	0.0	3	1.7	13	1.0	
FC questionnaire/Standing up assessment													p<0.001
Slow	12	0.8	1	5.9	0	0.0	0	0.0	1	0.6	10	0.8	
Somewhat slow	349	23.1	4	23.5	7	38.9	16	41.0	45	25.6	277	22.0	
Normal	272	18.0	2	11.8	3	16.7	6	15.4	34	19.3	227	18.0	
Somewhat fast	591	39.2	7	41.2	5	27.8	12	30.8	71	40.3	496	39.4	
Fast	243	16.1	1	5.9	2	11.1	4	10.3	16	9.1	220	17.5	
Not performed	41	2.7	2	11.8	1	5.6	1	2.6	9	5.1	28	2.2	
FC questionnaire Finger-ring frailty test													p=0.014
A gap is present	187	12.4	1	5.9	1	5.6	4	10.3	17	9.7	164	13.0	
An exact circle	657	43.6	6	35.3	7	38.9	13	33.3	76	43.2	555	44.1	
Cannot make a complete circle due to the thickness of muscles	660	43.8	10	58.8	10	55.6	22	56.4	83	47.2	535	42.5	
Not performed	4	0.3	0	0.0	0	0.0	0	0.0	0	0.0	4	0.3	
FC questionnaire Mastication													p=0.004
Green	17	1.1	1	5.9	0	0.0	2	5.1	4	2.3	10	0.8	
Yellow-green	157	10.4	2	11.8	4	22.2	6	15.4	24	13.6	121	9.6	
Pink	448	29.7	4	23.5	2	11.1	8	20.5	44	25.0	390	31.0	
Dark cerise	461	30.6	3	17.6	4	22.2	5	12.8	49	27.8	400	31.8	
Red	325	21.6	1	5.9	1	5.6	8	20.5	29	16.5	286	22.7	
Not performed	100	6.6	6	35.3	7	38.9	10	25.6	26	14.8	51	4.1	
FC questionnaire Swallowing													p=0.462
Less than 2 times	27	1.8	0	0.0	1	5.6	0	0.0	2	1.1	24	1.9	
More than 3 times	1,463	97.0	17	100.0	17	94.4	39	100.0	169	96.0	1,221	97.1	
Not performed	18	1.2	0	0.0	0	0.0	0	0.0	5	2.8	13	1.0	

**Table 4:** Data pertaining to items in the EQ-5D questionnaire that demonstrated association with the number of remaining teeth EQ-5D, European Quality of Life Five Dimension; VAS, visual analog scale; SD, standard deviation.

		Total	Number of teeth (n)					Jonckheere-Terpstra test
			0	1-4	5-9	10-19	>20	
EQ-5D utility index	N	1,414	14	15	34	162	1,189	
	Mean	0.92	0.81	0.91	0.87	0.90	0.92	p=0.002
	SD	0.10	0.25	0.12	0.17	0.12	0.10	
EQ-5D VAS	N	1,416	16	15	35	164	1,186	
	Mean	80.4	72.9	77.0	75.9	77.4	81.1	p<0.001
	SD	13.5	21.5	13.5	19.4	13.7	13.0	
EQ5D 1 - Degree of mobility	N	1,430	15	15	36	164	1,200	
	Mean	1.16	1.53	1.20	1.36	1.28	1.13	p<0.001
	SD	0.50	1.06	0.56	0.80	0.67	0.44	
EQ5D 2 - Personal care	N	1,432	16	15	35	164	1,202	
	Mean	1.03	1.50	1.00	1.11	1.03	1.02	p=0.044
	SD	0.24	1.10	0.00	0.53	0.20	0.19	
EQ5D 3 - Regular activities	N	1,434	16	16	36	164	1,202	
	Mean	1.08	1.31	1.44	1.25	1.12	1.06	p<0.001
	SD	0.35	0.70	1.09	0.65	0.43	0.29	
EQ5D 4 - Pain/discomfort	N	1,430	15	15	36	165	1,199	
	Mean	1.54	1.93	1.53	1.75	1.69	1.51	p=0.004
	SD	0.71	1.03	0.74	0.97	0.85	0.66	
EQ5D 5 - Anxiety/melancholy	N	1,424	16	16	35	162	1,195	
	Mean	1.18	1.63	1.31	1.29	1.21	1.17	p=0.010
	SD	0.46	1.02	0.48	0.52	0.50	0.44	

## Discussion

The present study used baseline data from a prospective study performed under the Kobe project to explore new strategies for reducing the social burden of dementia in Japan [4]. The results from this cohort indicated a significant increase in long-term care or support needs, with a reduction in the number of remaining teeth among healthy older adults in Kobe City; a similar trend was also observed for long-term care needs.

The findings also suggested that a reduction in the number of remaining teeth was associated with general frailty, including motor and oral functions, prevention of seclusion, memory, mental health, mastication scores, and the total number of food items that could be chewed. BMI was also significantly associated with the number of remaining teeth, indicating the impact of poor nutrition on declining function.

Previous studies demonstrated an association between tooth loss and functional disabilities in older adults. A large prospective study from Australia, which used the EQ-5D-5L to evaluate the extent of disability, demonstrated an independent effect of self-reported oral health on responses to the questionnaire, indicating the impact of oral health problems on general health. The authors noted that oral symptoms were related to compliance with dietary guidelines, which in turn were associated with health status [9]. In our cohort, an association was observed between the number of remaining teeth and oral function, mastication scores, and the number of food items that could be chewed. These findings reinforce the need for nutritional screening and intervention in this population.

Numerous studies have reported an association between tooth loss and functional disability in older adults. A large prospective cohort study performed among community-dwelling adults in Japan, who were aged at least 70 years, showed a significantly increased risk of functional disability in those with fewer remaining teeth [10]. The authors concluded that regular dental care may reduce the risk of functional disability in older adults with missing teeth. Another prospective study performed under the same project investigated the association between maximum occlusal force, an objective predictor of chewing ability, and incident functional disability in Japanese adults aged at least 70 years. The findings demonstrated that a decreased maximum occlusal force conferred an increased risk of functional disability even after adjusting for possible confounding factors; this effect was observed irrespective of the number of remaining teeth. Nevertheless, these findings indicate the importance of maintaining oral function; they also demonstrate the association between the state of oral and general health in the elderly [11].

A recent prospective cohort study in Japan that evaluated the association between the number of teeth and functional disability in community-dwelling older adults found that having fewer than 20 teeth was an independent risk factor for functional disability; this association persisted after propensity score matching [5]. The authors reported that dementia, stroke, frailty, and other systemic conditions were the major causes of long-term care certification, which was the primary endpoint of this study. In this context, changes in nutrition and malnutrition have been reported as risk factors for the development of dementia, stroke, and frailty [15-17]. The number of teeth, therefore, plays a vital role in preventing conditions that require long-term care.

In the context of QOL, a study using data from the Korean National Health and Nutrition Examination Survey conducted over two years between 2010 and 2012 employed the EQ-5D-5L tool to evaluate the relationship between the number of remaining teeth and health-related QOL. The findings from this large dataset indicated that subjects who had more teeth had increased QOL scores; high QOL was particularly associated with certain components of the EQ-5D-5L, including mobility, self-care, and daily living [12]. Our findings also indicate a definite association between the number of teeth and motor function.

Cognitive impairment is a major contributor to the nursing care needs of older adults. In view of the likelihood of tooth loss in this population, a prospective cohort study from Japan that extended over four years evaluated the association between tooth loss and cognitive impairment in older adults living in the community. The subjects in this study were of a similar age group as that of our cohort. However, the authors used Mini-Mental State Examination scores to determine subjects’ cognitive function. These findings suggest that tooth loss was a predictor of cognitive decline in this cohort and that both tooth loss and cognitive impairment chronically accumulated over time [6]. In our cohort, significant associations were observed between the number of remaining teeth, memory loss, and mental health status.

The major limitation of the present study is the sample size. Although the number of subjects was adequate for a reliable estimation of association, the level of significance of the association with individual items on the Frail Check and EQ-5D-5L questionnaires and Frail Check tests might have been affected. In addition, our cohort comprised individuals aged 64-65 years, which is a relatively young age group for assessing long-term care needs.

Further large-scale prospective studies with a longitudinal design are needed to address these limitations, including adults from older age groups. Despite these limitations, the findings of the present analysis indicate that tooth loss has a significant impact on the long-term care and support needs of community-dwelling older adults.

## Conclusions

This study found a significant association between the number of remaining teeth and the need for long-term care or support in a group of community-dwelling older adults aged 64-65 years. These findings emphasize the importance of maintaining oral health and highlight the potential impact of tooth loss on overall health and functional abilities. Further research with larger cohorts and older age groups is warranted to validate these findings and better understand the implications of tooth loss in long-term care.
